# Inducible nitric oxide synthase links NF-κB to PGE_2 _in polyunsaturated fatty acid altered fibroblast *in-vitro *wound healing

**DOI:** 10.1186/1476-511X-4-14

**Published:** 2005-07-12

**Authors:** Yi Jia, John J Turek

**Affiliations:** 1 Department of Basic Medical Sciences, Purdue University, West Lafayette, Indiana 47907, USA

## Abstract

**Background:**

This study investigated mechanisms of altered fibroblast collagen production induced by polyunsaturated fatty acids. 3T3-Swiss fibroblasts were grown in medium containing either eicosapentaenoic or arachidonic acid. The effects of nuclear factor-kappaB activation by lipopolysaccharide on inducible nitric oxide synthase, nitric oxide, prostaglandin E_2_, collagen production, and *in-vitro *wound healing were studied.

**Results:**

Eicosapentaenoic acid treated cells produced less prostaglandin E_2 _but had increased inducible nitric oxide synthase expression, nitric oxide production, collagen formation, and recoverage area during *in-vitro *wound healing than cells treated with arachidonic acid. Activation of nuclear factor-kappaB with lipopolysaccharide increased inducible nitric oxide synthase expression, the production of nitric oxide, prostaglandin E_2_, collagen, and the *in-vitro *wound recoverage area. The nitric oxide synthase inhibitor, N^G^-nitro-*L*-arginine methyl ester, decreased lipopolysaccharide-induced nitric oxide, but the amount of nitric oxide was greater in eicosapentaenoic acid treated cells. N^G^-nitro-*L*-arginine methyl ester plus lipopolysaccharide treatment increased collagen production and cellular recoverage area while treatment with N^G^-nitro-*L*-arginine methyl ester alone decreased it in wounded fibroblasts.

**Conclusion:**

The activation of the NF-κB pathway and PGE_2 _can be linked by the cross-talk of iNOS and NO in the PUFA altered fibroblast collagen production and wound healing. Additional studies are needed to determine how polyunsaturated fatty acids can be used as adjuvants in combination with other treatments (i.e, drugs) to design therapies to either enhance healthy collagen production or inhibit production and reduce fibrosis.

## Background

The purpose of this research was to test the hypothesis that polyunsaturated fatty acids can alter collagen formation during *in-vitro *healing by changing iNOS expression and NO production, which have cross-interaction with the nuclear transcription factor kappaB (NF-κB) pathway and PGE_2_. The control of collagen formation for optimal healing in tissues and organs is essential for numerous diseases [1-3]. The healing of skin and connective tissues such as ligaments require enhanced and effective production of healthy collagen for strength and to shorten the recovery time, but without scarring. However, collagen formation in injured vital organs needs to be minimized to prevent fibrosis and subsequent loss of organ function. Both enhanced and reduced collagen formation likely have common regulatory mechanisms that remain to be elucidated. Multiple cellular and extracellular factors can influence collagen formation, such as nitric oxide [4], PGE_2 _[5], as well as growth factors [6] and matrix metalloproteinases [7]. Therefore, potential therapies to control healing may require an approach targeting multiple molecules or mechanisms.

Our previous studies showed that polyunsaturated fatty acids (PUFA) alter collagen production in avian chondrocytes [8], porcine medial collateral ligament fibroblasts [9], and murine 3T3-Swiss fibroblasts [5]. Eicosapentaenoic acid (EPA, 20:5 *n*-3) treated porcine medial collateral ligament fibroblasts produced more collagen than those treated with arachidonic acid (AA, 20:4 *n*-6) [9]. In murine 3T3-Swiss fibroblasts, we have observed that collagen production could be regulated by exposure to different *n*-6: *n*-3 PUFA ratios and these effects were mediated, in part, by PGE_2 _and changes in the signaling via the different PGE receptor subtypes [5].

Since many collagen formation associated genes have promoter or enhancer elements for NF-κB [10], we studied the different response of NF-κB related genes to EPA and AA treatments in 3T3-Swiss fibroblasts by a gene profiling system [11]. Treatments with lipopolysaccharide (LPS), an NF-κB inducer, stimulated increased expression of several genes in the NF-κB pathway that are linked collagen production (i.e, interleukin-6 and inducible nitric oxide synthase).

The activated NF-κB dimer binds to the 5'-flanking region of the iNOS promoter and induces iNOS formation [12]. Inducible nitric oxide synthase (iNOS) and nitric oxide (NO) have an important role in collagen formation during wound healing [13,14]. Inhibition of NO synthase significantly decreased collagen synthesis in wound fibroblasts [13]. Dermal fibroblasts from iNOS-knock out murine fibroblasts proliferated more slowly and synthesized less collagen, and NO donors restored the collagen synthesis to normal level [15]. Another study implicated a role for NO in PGE_2 _formation and collagen deposition in rats [16]. Therefore, endogenous iNOS and NO may link the NF-κB pathway to PGE_2 _and regulate collagen formation during wound healing.

## Results

### Real-time RT-PCR for iNOS mRNA

**Figure 1:**
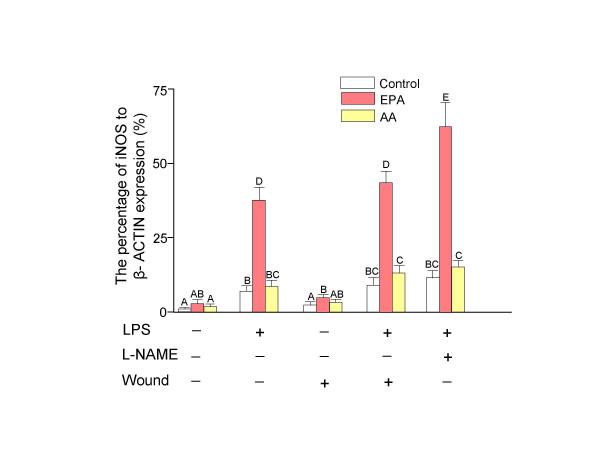
**Real-time RT-PCR for iNOS mRNA expression in 3T3-Swiss fibroblasts *n *= 3, ± SD). **The expression of iNOS mRNA can be altered by stimulation of the NF-κB pathway or inhibition of nitric oxide synthase in 3T3-Swiss fibroblasts. EPA treated fibroblasts were more sensitive to the changes of NF-κB pathway than AA treated cells. Cells were incubated for 48 hr with bovine serum albumin alone as control or bovine serum albumin-soap loaded fatty acids (25 μM). Medium was then replaced with fresh fatty acid enriched medium containing lipopolysaccharide (LPS, 10 μg/ml) with or without N^G^-nitro-*L*-arginine methyl ester (L-NAME, 10^-7 ^M) for another 24 hr. The wound was created after initial 48 hr treatments in duplicate set of plate with or without LPS. Cells were harvested at 24 hr post wounding and real-time RT-PCR was performed. The results were presented as the percentage of iNOS to β-actin mRNA expression. Bars with different letters are significantly different (*P *< 0.05). EPA, eicosapentaenoic acid; AA, arachidonic acid; LPS, lipopolysaccharide; L-NAME, N^G^-nitro-*L*-arginine methyl ester.

The transcriptional level of iNOS mRNA was determined by the real-time RT-PCR (Figure 1). The expression of iNOS mRNA can be altered by stimulation of the NF-κB pathway. Incubation with the NF-κB pathway inducer, lipopolysaccharide (LPS, 10 μg/ml), significantly (*P *< 0.01) increased the expression of iNOS mRNA in both arachidonic acid (AA, 20:4 *n*-6) and eicosapentanoic acid (EPA, 20:5 *n*-3) treated normal or wounded 3T3-Swiss fibroblasts. However, the EPA-treated fibroblasts were more responsive to induction of the NF-κB pathway than AA-treated cells. Activation of the NF-κB pathway in EPA-treated cells with LPS resulted in significantly increased (*P *< 0.001) transcription of iNOS mRNA compared to AA-treated cells. The addition of the nitric oxide synthase inhibitor, N^G^-nitro-*L*-arginine methyl ester (L-NAME, 10^-7 ^M) resulted in a significant increase (*P *< 0.05) in the transcription of iNOS mRNA only in EPA-treated fibroblasts with LPS stimulation.

### Quantification of nitrite

**Figure 2:**
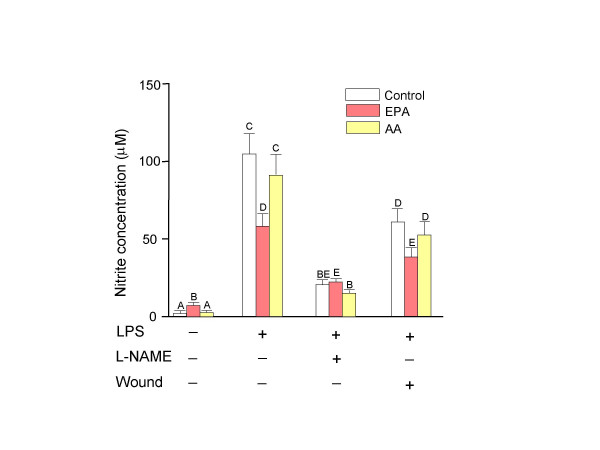
**NO production in 3T3-Swiss fibroblasts (n = 3, ± SD). **Cells were incubated for 48 hr with bovine serum albumin alone as control or bovine serum albumin-soap loaded fatty acids (25 μM). Medium was then replaced with fresh fatty acid enriched medium containing lipopolysaccharide (LPS, 10 μg/ml) with or without N^G^-nitro-*L*-arginine methyl ester (L-NAME, 10^-7 ^M) for another 24 hr. The wound was created after initial 48 hr treatments in a duplicate set of plate with LPS treatments. Culture supernatants (100 μl) were collected at 24 hr post wounding and quantification of nitrite was performed. The results were presented as the nitrite concentration (μM). Bars with different letters are significantly different (*P *< 0.05). EPA, eicosapentaenoic acid; AA, arachidonic acid; LPS, lipopolysaccharide; L-NAME, N^G^-nitro-*L*-arginine methyl ester.

A stable end product of NO synthesis, nitrite, was measured to determine the NO concentration (Figure 2). The production of nitrite can be changed by altering the activity of the NF-κB pathway and nitric oxide synthase. EPA-treated normal fibroblasts without LPS stimulation produced more nitrite than control or AA-treated fibroblasts. However, when stimulated by LPS, EPA-treated normal fibroblasts produced significantly (*P *< 0.05) less nitrite than control or AA-treated cells. Treatment with L-NAME significantly reduced LPS-induced nitrite production, and EPA-treated cells produced more nitrite than AA-treated cells. Wounded and LPS stimulated cells decreased nitrite production for all treatments compared to LPS alone, but EPA-treated fibroblasts produced less nitrite than AA-treated or control cells.

### Quantification of PGE_2_

**Figure 3:**
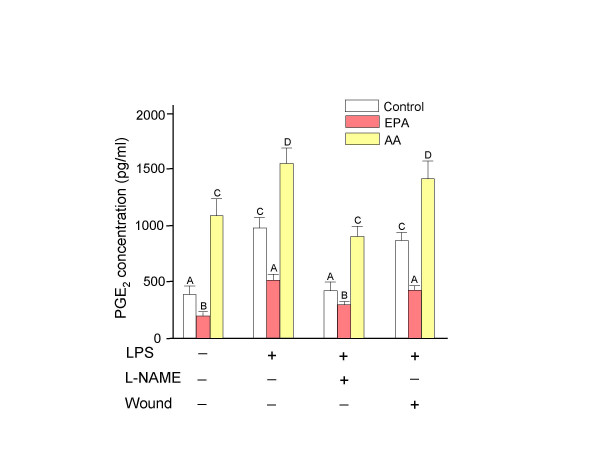
**PGE_2 _production in 3T3-Swiss fibroblasts *n *= 3, ± SD)**. The addition incubation of nitric oxide synthase inhibitor decreased the LPS induced PGE_2 _production in 3T3-Swiss fibroblasts. Cells were incubated for 48 hr with bovine serum albumin alone as control or bovine serum albumin-soap loaded fatty acids (25 μM). Medium was then replaced with fresh fatty acid enriched medium containing lipopolysaccharide (LPS, 10 μg/ml) with or without N^G^-nitro-*L*-arginine methyl ester (L-NAME, 10^-7 ^M) for another 24 hr. The wound was created after initial 48 hr treatments in duplicate set of plate with or without LPS. Culture supernatants were collected at 24 hr post wounding and the quantification of PGE_2_performed. The results were presented as the PGE_2 _concentration (pg/ml). Bars with different letters are significantly different (*P *< 0.05). EPA, eicosapentaenoic acid; AA, arachidonic acid; LPS, lipopolysaccharide; L-NAME, N^G^-nitro-*L*-arginine methyl ester.

Activation of NF-κB pathway by LPS in 3T3-Swiss fibroblasts significantly increased (p < 0.05) the production of PGE_2 _over control levels for all groups (Figure 3). Both basal and LPS stimulated amounts of PGE_2 _were lowest in EPA-treated fibroblasts compared to the control and AA-treated cells. The addition of the nitric oxide synthase inhibitor, L-NAME, to LPS stimulated fibroblasts decreased PGE_2 _production to basal levels for all groups. Wounding of LPS stimulated cells did not significantly change the amount of PGE_2 _produced by cells when compared with those stimulated by LPS alone.

### Collagen formation

Wounded fibroblasts without any PFA treatment increased both collagen production (CP) and collagen as a percentage of total proteins (C-PTP) compared to control (non-wounded, no PFA treatment) fibroblasts (Table 2). However, wounded fibroblasts treated with LPS produced less collagen and C-PTP than the control cells with LPS stimulation. Treatment with L-NAME alone decreased collagen production in control fibroblasts, while the additional of L-NAME plus LPS produced more collagen and had more collagen as a percentage of total protein in both control and wounded fibroblasts. The combination treatments of EPA, L-NAME and LPS produced higher CP and C-PTP than treatments of AA, L-NAME and LPS in normal fibroblasts.

**Table 2:** Collagen formation in 3T3-Swiss fibroblasts (n = 3, ± SD). Cells were incubated for 48 hr with bovine serum albumin alone as control or bovine serum albumin-soap loaded fatty acids (25 μM). The wound was then created and the medium was then replaced with fresh fatty acid enriched medium containing 50 μM ascorbic acid and 5 μCi of 3H-proline with or without lipopolysaccharide (LPS, 10 μg/ml) and N^G^-nitro-*L*-arginine methyl ester (L-NAME, 100 nM). A duplicate set of plates was made without wounding. Cells were harvested at 24 hr post wounding and assayed for collagen, total protein and DNA. The amount of collagen is expressed as DPM from ^3^H-proline per μg of DNA. Mean values within rows having different superscripts are significantly different (P < 0.05) by 1-way ANOVA and Tukey test. EPA, eicosapentaenoic acid; AA, arachidonic acid; LPS, lipopolysaccharide; L-NAME, N^G^-nitro-*L*-arginine methyl ester.

Treatments	Collagen production (CP) (DPM/μg DNA amount)	Collagen as a percentage of total protein (C-PTP) (%)
Control	2419.6 ± 318.1^a^	18.7 ± 1.6^a^
Wound	3044.8 ± 269.2^b^	21.8 ± 1.5^b^
LPS	3953.3 ± 57.3^c^	26.5 ± 0.8^c^
L-NAME	1225.6 ± 270.7^d^	11.4 ± 2.7^d^
LPS + Wound	2650.3 ± 146.9^ab^	19.0 ± 1.8^ab^
LPS + L-NAME	5393.7 ± 583.4^e^	37.5 ± 3.3^e^
LPS + L-NAME + EPA	6580.2 ± 418.3^e^	42.4 ± 5.6^e^
LPS + L-NAME + AA	3511.3 ± 561.4^c^	26.7 ± 4.0^c^

### In-vitro wounding assay

**Figure 4:**
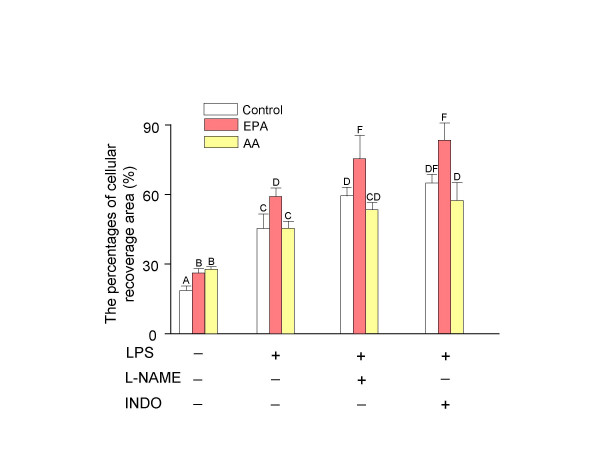
***In-vitro *wounding assay in 3T3-Swiss fibroblasts (n = 3, ± SD). **The addition treatment of L-NAME to LPS increased the percentages of cellular recover area while the treatment of L-NAME alone decreased it in wounded 3T3-Swiss fibroblasts. When stimulated with LPS, EPA treated fibroblasts had higher the percentage of cellular recover area than the AA treated cells. Cells were incubated for 48 hr with bovine serum albumin alone as control or bovine serum albumin-soap loaded fatty acids (25 μM). Then wound was created and the medium was replaced with fresh fatty acid enriched medium containing lipopolysaccharide (LPS, 10 μg/ml), N^G^-nitro-*L*-arginine methyl ester (L-NAME, 10^-7 ^M) and indomethacin (10^-8 ^M) for another 24 hr. Multiple photographs of the wound were obtained and the percentage of cellular recover areas were determined. Bars with different letters are significantly different (P < 0.05). EPA, eicosapentaenoic acid; AA, arachidonic acid; LPS, lipopolysaccharide; L-NAME, N^G^-nitro-*L*-arginine methyl ester; INDO, indomethacin.

The cellular recoverage area in wounded fibroblasts changed by the treatments of LPS, nitric oxide synthase inhibitor, cyclooxygenase (COX) inhibitor, and PFA (Figure 4). After 24 hr post wounding, LPS activation of the NF-κB pathway increased the percentage of cellular recoverage area compared to the control. The treatment of L-NAME alone decreased the percentage of cellular recoverage area in all groups while the treatment of L-NAME plus LPS increased the cellular recoverage area compared to LPS treatment alone. Administration of indomethacin, a cyclooxygenase inhibitor, increased the cellular recoverage area in LPS stimulated fibroblasts. The treatments of EPA and AA alone both increased the recoverage area compared to the control. EPA-treated fibroblasts with LPS had a higher the percentage of cellular recoverage area than the AA plus LPS treated cells, with or without other treatments.

## Discussion

Polyunsaturated fatty acids affect the formation of numerous mediators of inflammation, such as eicosanoids and cytokines [9]. Thus, dietary fatty acids, those mobilized from cellular phospholipids or triacylglycerols stored in adipose tissue, and topical applications [18] can all potentially influence wound healing. A limited number of studies have examined the effects of dietary or topical applications of PUFA on wound healing. A cutaneous wound healing study found that rats fed a diet enriched in *n*-3 fatty acids produced wounds that were weaker in tensile strength compared to those from rats fed a diet containing *n*-6 fatty acids [19]. Cutaneous wounds in dogs fed an *n*-3 enriched diet had reduced epithelialization and contraction of open wounds and less edema in sutured wounds after 5 days than dogs fed a diet enriched in *n*-6 fatty acids. However, the *n*-3 diet did not appear to have a negative effect on wound healing [20]. A more recent study found that topical application of oleic acid (monounsaturated *n*-9 fatty acid) to surgically induced skin wounds in mice resulted in more rapid wound closure than applications of linolenic acid (18:3, *n*-3) or linoleic acid (18:2, *n*-6) [18]. The topical application of linolenic acid also resulted in an increased amount of connective tissue fibers compared to other fatty acid treatments. These limited studies suggest that in order to take advantage of the inflammation mediating properties of PUFA and use them as adjuvants in the healing process, it may be necessary to use the different classes of PUFA selectively during the stages of wound healing. Wound healing consists of an inflammatory phase, a proliferative (fibroblastic) phase, and a remodeling (maturation) phase [21,22]. In addition, the type of wound (e.g., acute or chouronic) and patient (e.g., diabetic, burn injured, or immunosuppressed) will require matching the appropriate PUFA to the particular stage of wound healing. For example, in immunosuppressed patients, a PUFA that enhances inflammation in the early stages may be needed to stimulate the healing process.

**Figure 5:**
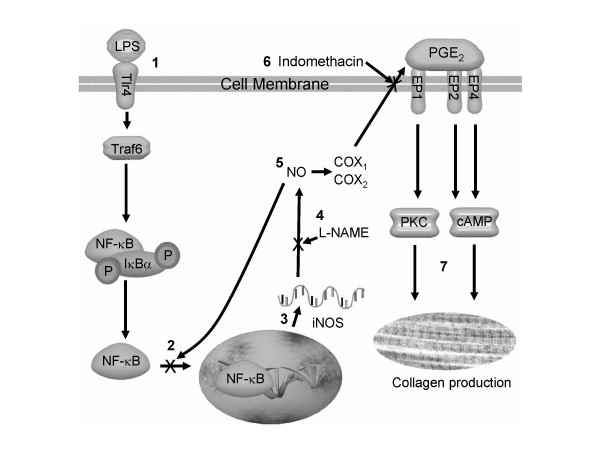
**Proposed model for regulation of collagen formation in these experiments. **1. Activation of the nuclear factor-κB (NF-κB) signaling pathway by lipopolysaccharide. 2. NO can inhibit the binding of NF-κB family members to target DNA. 3. NF-κB family binding to DNA and transcription of iNOS mRNA. 4. L-NAME inhibits the production of NO from iNOS. 5. NO regulates PGE_2 _production divergently through activation of COX isoforms. 6. Indomethacin inhibits COX and reduces PGE_2_production. 7. Collagen production and wound healing processes are changed by the activation of different PGE_2 _receptors subtypes and second messengers. LPS, lipopolysaccharide; Tlr4, Toll-like receptor 4; Traf6, Tumor necrosis factor receptor-associated factor 6; IκB, Inhibitor of kappa light polypeptide gene enhancer in B-cell; NF-κB, nuclear transcription factor kappaB; iNOS, inducible nitric oxide synthase; L-NAME, NG-nitro-L-arginine methyl ester; NO, nitric oxide; COX, cyclooxygenase; INDO, indomethacin; PKC, protein kinase C; cAMP, cyclic adenosine monophosphate.

A component of optimal healing is regulation of collagen formation. Favorable collagen formation includes both enhanced collagen production for healing connective tissues and diminished formation in vital organs to minimize fibrosis. Our previous studies revealed that polyunsaturated fatty acids affected a number of mediators and pathways involved in collagen formation [5,9]. Many collagen formation associated genes, such as IL-6 and iNOS, have promoter or enhancer elements for NF-κB [23,24]. These genes provide ideal targets to connect various pathways involved in PUFA influenced collagen formation. The present paper studied the link between NF-κB pathway generated iNOS and nitric oxide (NO) in PUFA altered collagen formation, and the association with PGE_2 _production. Our hypothesis in the present study is that polyunsaturated fatty acids can alter collagen formation during *in-vitro *healing by changing iNOS expression and NO production, which have cross-interaction with the NF-κB pathway and PGE_2 _(Figure 4, 5).

NO is synthesized from the terminal guanidine nitrogen atom of *L*-arginine by nitric oxide synthase (NOS) and involved in many biological functions [25]. The inducible isoform of NOS (iNOS) is regulated primarily at the transcriptional level and contributes to most of the NO produced compared to the other isoforms [26]. The NF-κB signaling pathway regulates promoter regions and induces iNOS transcription [12]. In the present study using real-time RT-PCR, activation of the NF-κB pathway by LPS increased the transcription of iNOS mRNA in 3T3-Swiss fibroblasts. The increase was greatest in EPA-treated fibroblasts. Other researchers reported that NO can also regulate iNOS transcription through a negative feedback by inhibiting NF-κB binding to DNA [27]. In our experiments, the addition of a small amount of the nitric oxide synthase inhibitor, L-NAME, increased the transcription of iNOS mRNA only in EPA plus LPS treated normal fibroblasts. However, the increased iNOS expression in EPA plus LPS treated cells did not result in increased nitric oxide (nitrite) production, and EPA plus LPS treated cells produced less nitric oxide than control or AA treated cells. The decreased amount of nitric oxide with EPA plus LPS treatment may enhance iNOS expression due to decreased negative feedback on NF-κB binding to DNA (Figure 4, 5). The observation that increased iNOS expression in EPA-treated cells does not lead to increased NO production, may be explained by altered post-transcriptional regulation that leads to reduced synthesis of iNOS protein and thus decreased NO production.

Reduced NO in EPA treated cells, even though there is increased transcription of iNOS mRNA, can also be linked to PGE_2 _production in these cells. The amount of PGE_2 _produced by the 3T3 fibroblasts was inversely related to the amount of nitric oxide produced. PGE_2 _will bind to PGE receptors (EP1, EP2, EP3, and EP4) that activate signaling by protein kinase C (PKC) or cyclic AMP (cAMP). Cells treated with AA are mainly responsive to signaling from the EP1 receptor (PKC signaling), whereas EPA-treated cells are mainly responsive to EP2 and EP4 signaling (cAMP signaling) [5]. Arachidonic acid alone has been shown to function as a secondary messenger and activate PKC. The increased amount of PGE_2 _in AA-treated cells and stimulation of the PKC signaling pathway via the EP1 receptor (e.g., PKC activation) could affect post-translational synthesis of iNOS protein. Other studies identified a role for PKC in the induction of iNOS by the NF-κB pathway in murine 3T3 fibroblasts [28] and the nitric oxide action on angiogenesis [29].

Nitric oxide regulates PGE_2 _production in a divergent manner. Nitric oxide stimulated PGE_2 _release in macrophages [30,31] while endogenous NO inhibited PGE_2 _production in LPS-stimulated macrophages [32]. The dual effect of NO on PGE_2 _production may be due to the different effects on cyclooxygenase (COX) isoforms. Study of COX isoform deficient murine fibroblasts revealed that NO changed PGE_2 _production by activating COX-1 but inhibiting COX-2 [30]. Other studies showed that low concentrations of nitric oxide attenuate PGE_2 _production induced by LPS, in part, due to decreased expression of COX-2 protein [33]. However, LPS activation of the NF-κB pathway can influence COX-2 gene expression by directly altering COX-2 mRNA transcription [34]. Our results showed that inhibition of NO with L-NAME, decreased LPS-induced PGE_2 _production in normal 3T3-Swiss fibroblasts. Thus, PGE_2_production in 3T3-Swiss fibroblasts may be regulated at the COX-2 transcription level through the NF-κB pathway or the COX-2 post-translational level through the production of NO derived via nitric oxide synthase. The combination of PUFA treatment (EPA) plus blocking of nitric oxide production with L-NAME, resulted in a synergistic effect that enhanced collagen production compared to control fibroblasts.

The suppression of type I collagen gene expression by PGE_2 _can be mediated by both altering the amount and steady-state of collagen mRNA [35,36]. Interestingly, inhibition of iNOS activity has been shown to both slow collagen production in the healing process and also favor collagen deposition and development of fibrosis [37]. It was hypothesized that NO scavenged reactive oxygen species by formation of peroxynitrite, and iNOS inhibition blocked NO formation and contributed to the development of fibrosis [37]. Therefore, appropriate induction of iNOS from the NF-κB signaling pathway may be a critical mechanism for controlling collagen formation in both fibrosis and proper wound healing.

Changes in collagen formation have also been correlated to the concentration of NO produced. Collagen production (CP) and collagen as a percentage of total protein (C-PTP) was increased in LPS and interferon-γ (IFN-γ) incubated wound derived murine fibroblasts [13]. Additional treatments of NO synthase inhibitors decreased the CP and C-PTP in the same study. However, LPS and IFN-γ together, decreased, while inhibitors of NO restored the collagen production in human small intestinal lamina propria fibroblasts [38]. Another study in normal rat skin fibroblasts showed that a nitric oxide donor at low concentration enhanced, but at higher concentration reverted collagen synthesis back to control levels [4]. Dermal fibroblasts from iNOS-knock out mice proliferated more slowly and synthesized less collagen [15], and transfection with iNOS enhanced the collagen production in a cutaneous wounded rat model [39]. In our experiments, LPS stimulated the expression of iNOS mRNA, NO production, and collagen synthesis in 3T3-Swiss fibroblasts. The additional incubation of small amount of L-NAME to LPS decreased the NO production to a low level but increased the expression of iNOS mRNA and the collagen production.

Healing fibroblasts are phenotypically characterized by changes in collagen production, cell proliferation, and migration. Wound cells have been shown to increase the expression of iNOS and production of NO in various models [13,40]. Inhibition of iNOS delayed the reepithelialization in cutaneous wound repair [41]. The delayed wound repair in iNOS knockout mice was reversed by iNOS gene transfer [42]. Interestingly, the role of nitric oxide on cell proliferation after wounding is concentration-dependent [4]. At low concentration, NO promoted cell proliferation in murine fibroblasts [43], while at higher concentrations, NO decreased the proliferation of rat dermal skin fibroblasts [4]. The present study showed that the treatment of L-NAME alone inhibited the NO production and decreased the percentage of cellular recoverage area in 3T3-Swiss fibroblasts. However, the additional treatment of L-NAME to fibroblasts stimulated by LPS increased cell proliferation and migration compared to the LPS treatments alone. L-NAME alone may block the effect of nitric oxide at low concentration while the addition of L-NAME plus LPS decreased the LPS induced NO to a low concentration and increased the collagen formation, cell proliferation, and migration. Indomethacin, a cyclooxygenase inhibitor, increased the percentage of cellular recoverage area in fibroblasts with LPS stimulation. The decreased PGE_2 _induced by indomethacin also stimulated wound healing in 3T3-Swiss fibroblasts.

In summary, we demonstrated that iNOS and NO provide a cross-talk between the activation of the NF-κB pathway and PGE_2 _in the PUFA altered fibroblast collagen production and wound healing. The use of PUFA for regulation of collagen formation in wound healing or fibrosis will require additional experiments in order to determine how they may be used as adjuvants. Most likely it will be necessary to use a particular class of fatty acids (e.g., *n*-3, *n*-6, *n*-9) appropriate to the type of wound and stage of wound healing. In addition, use of fatty acids in combination with selective targeting of transcription factors and mediators (e.g., stimulation or inhibition) that regulate collagen formation will provide the level of control necessary to adapt the treatment protocol to the type of wound.

## Methods

### Reagents

Reagents were purchased from Sigma-Aldrich, St. Louis, MO, unless specified otherwise.

### Cell culture and fatty acid enrichment

Mouse fibroblast (3T3-Swiss albino; American Type Culture Collection CCL-92, Rockville, MD) were maintained as subconfluent monolayers in six-well plates (Corning Costar, Cambridge MA) with Dulbecco's modified Eagle's medium (DMEM), 4 mM L-glutamine, 1.5 g/L sodium bicarbonate, 4.5 g/L glucose, and 10% bovine calf serum (Hyclone, Logan, UT). Subconfluent cultures grown for 24 hr in maintenance medium were washed twice and changed to fresh medium minus calf serum. In place of serum, the control medium was supplemented with 5 mg/100 ml of fatty acid free bovine serum albumin (BSA); and the fatty acid enriched test media were supplemented with BSA-loaded fatty acid soaps of arachidonic acid (AA, 20:4 *n*-6) or eicosapentaenoic acid (EPA, 20:5 *n*-3) (Nu-Chek Prep, Elysian, MN) to a final concentration of 25 μM. Cells were grown for 48 hr in the test media prior to addition of treatments described below.

### In vitro wound assay

An *in vitro *wound healing assay was used to evaluate the migration and proliferation of 3T3-Swiss fibroblast as previous studies [9]. Briefly, a sterile pipette tip was used to make a 0.5-mm-wide wound by streaking across a monolayer of 3T3 Swiss fibroblasts. The wound was created when the cells were about 80% confluent after the initial 48 hr polyenoic fatty acid (PFA) enrichment and the migration of fibroblasts into the wound was measured at 24 hr post wounding. Multiple photographs of the wound were obtained by phase contrast microscopy, and the mean areas of cell recoverage for each sample were determined with image analysis software (Optimas 6.1, Media Cybernetics, Silver Spring, MD). The percentage of cellular recoverage area to the whole wound area was measured to evaluate the combined effects of cell proliferation and migration.

A similar set of plates was used to perform the real-time RT-PCR of iNOS, quantification of nitrite and PGE_2_, and collagen synthesis. For real-time RT-PCR and the quantification of nitrite and PGE_2_, the following protocol was used. After the initial 48 hr PFA enrichment, the media was changed with fresh fatty acid enriched medium containing *Eshericia coli *O55:B5 lipopolysaccharide (LPS, 10 μg/ml) with or without N^G^-nitro-*L*-arginine methyl ester (L-NAME, 10^-7 ^M) and indomethacin (INDO, 10^-8 ^M). Lipopolysaccharide is used to activate the NF-κB pathway and mimic some aspects of *in vivo *inflammation. For collagen formation, after the initial 48 hr polyenoic fatty acid (PFA) enrichment, the media was replaced with fresh fatty acid enriched medium containing 50 μM ascorbic acid and 5 μCi of ^3^H-proline (Amersham, Arlington Heights IL) with or without treatments. At 24 hr post wounding, a portion of the media was collected or the cells were harvested. Parallel plates were also cultured with the same treatments but without wounding. The cells for all treatments used in these experiments had greater than 99% viability based upon the standard trypan blue dye exclusion test.

### RNA isolation and cDNA synthesis

After cells were washed with Hank's balanced salt solution and harvested, total RNA was isolated using a commercially available kit (RNAqueous, Ambion, Austin, TX) and digested with RNase-free DNase, as recommended by the supplier. The concentration and purity of total RNA were determined by measuring the optical density at 260 and 280 nm. The ratio of absorbance at 260 to 280 nm was 1.8–2.0. Two μg total RNA was reverse transcribed using a cDNA synthesis Kit (iScript™ cDNA Synthesis Kit, Bio-Rad Laboratories, Hercules, CA) following the manufacture's instructions. Generated cDNA were diluted with RNA-free water before usage.

### Real-time RT-PCR

Synthesized cDNA encoding for murine iNOS and β-actin (as endogenous control) were amplified and analyzed by a real-time reverse transcription polymerase chain reaction (real time RT-PCR) system (Applied Biosystems 7300 Real Time PCR System, Foster City, CA). The oligonucleotide primers were designed by Primer Express software (Primer Express 2.0, Applied Biosystems) and ordered from IDT (Integrated DNA Technologies, Coralville, IA). The cDNA sequences were obtained from the Genebank database as indicated in table 1. PCR amplifications were performed according to the manufacture's protocol. Samples were prepared in a total volume of 50 μl containing 2 μl cDNA sample (diluted 1x, 10x, 100x, 200x), 25 μl SYBR Green PCR Master Mix (Applied Biosystems, Foster City, CA), 1 μl each primer, and 21 μl RNA-free water. For each reaction, the polymerase was initiated at 50°C for 2 min and 95°C for 10 min, and amplification was then performed at 35 cycles of switching between 95°C for 15 seconds, 55°C for 30 seconds, and 72°C for 15 seconds followed by melting point analysis from 60°C to 95°C. All samples were done in triplicate and the coefficient was determined by creating a standard curve from plotting C_T _values vs. the total RNA. The results were presented as the mRNA expression percentage of iNOS to β-actin.

**Table 1:** Primers for iNOS and β-actin. Primers for iNOS and β-actin were designed by Primer Express^®^2.0 (Applied Biosystems). iNOS, inducible nitric oxide synthase.

Gene	Genebank accession #	Start positions	Primer sequences (5' ? 3')	Amplicon length (bp)
iNOS	U43428	2407	AGGGAATCTTGGAGCGAGTTGT	103
		2509	AGCCTCTTGTCTTTGACCCAGT	
β-actin	X03672	886	TGGAATCCTGTGGCATCCATGA	91
		976	AGCACTGTGTTGGCATAGAGGT	

### Quantification of nitrite

Nitrite (NO2-) in culture supernatants was measured to determine the synthesis of NO as other studies [17]. Culture supernatants (100 μl) were mixed with an equal volume of Griess reagent (1% sulfanilamide, 0.1% naphthylethylene diamine dihydrochloride and 2.5% phosphoric acid) and incubated at the room temperature with dimmed light for 10 min. Nitrite was measured at 550 nm by a microplate reader (EIA reader 2550, Bio-Rad, Hercules, CA) using the double-distilled water as blank and the sodium nitrite to generate a standard curve.

### Quantification of PGE_2_

Assessment of total prostaglandin E_2 _(PGE_2_) amount from the medium of 3T3-Swiss fibroblasts incubation was preformed by an ELISA kit (Monoclonal prostaglandin E_2 _EIA Kit-514010; Cayman Chemical, Ann Arbor, MI), according to the manufacturer's recommended protocol.

### Collagen assay

Collagen was assayed as described previously [5]. The media was collected and the cells washed twice with cold phosphate buffered saline (PBS). The cells were pelleted by centrifugation and the PBS wash combined with the media fraction. The cell pellet was suspended in 1.0 ml of ammonium hydroxide-Triton X-100 cell lysing solution (AT solution). Following 15-minute incubation at 37°C, 750 μl of the lysate is combined with the media fraction. The two fractions, cell and media, then underwent trichloroaceticic acid (TCA) precipitation (equal volume of 20% TCA added to the cell plus media fraction). The acid insoluble precipitate was then rinsed several times with 10% TCA to remove free ^3^H-proline. The precipitate was redissolved in 0.05 N NaOH in 0.05 M TES buffer plus 0.005 M CaCl_2 _and half of the solution incubated for 6 hr at 37°C with protease-free Type VII collagenase (100 U/ mL) in TES and the other half of the solution served as a control. Following the digestion, TCA was again added to precipitate the acid insoluble proteins; however, the collagen fragments generated by collagenase treatment remain in solution. The supernatant and precipitate were counted in a scintillation counter and collagen, non-collagenous, and total protein production reported as DPM/μg DNA.

### DNA assay

The remaining 250 μl of the AT solution cellular-lysate from the collagen synthesis assay was used for total DNA determination. Picogreen, 50 μl (Molecular Probes, Eugene OR) was added to 50 μl of lysate or to 50 μl of known DNA standards and fluorescence of the dye binding to double stranded DNA was measured in a spectofluorometer. A DNA standard curve was generated by linear regression and sample DNA values was used to obtain the unknown values.

### Statistical analysis

Data were presented as means ± standard deviation (SD) and analyzed by both one-way and two-way ANOVA procedures of SAS (SAS Institute, Cary, NC). A Tukey test was used to analyze significant main and interaction effects. A *P *value < 0.05 was considered statistically significant.
